# Supplementary figures and images for: A Novel Fibromodulin Antagonist Peptide RP4 Exerts Antitumor Effects on Colorectal Cancer
Source: Pharmaceutics. 2023 Mar 14;15(3):944. doi: 10.3390/pharmaceutics15030944 (PMC10053243; doi:10.3390/pharmaceutics15030944)

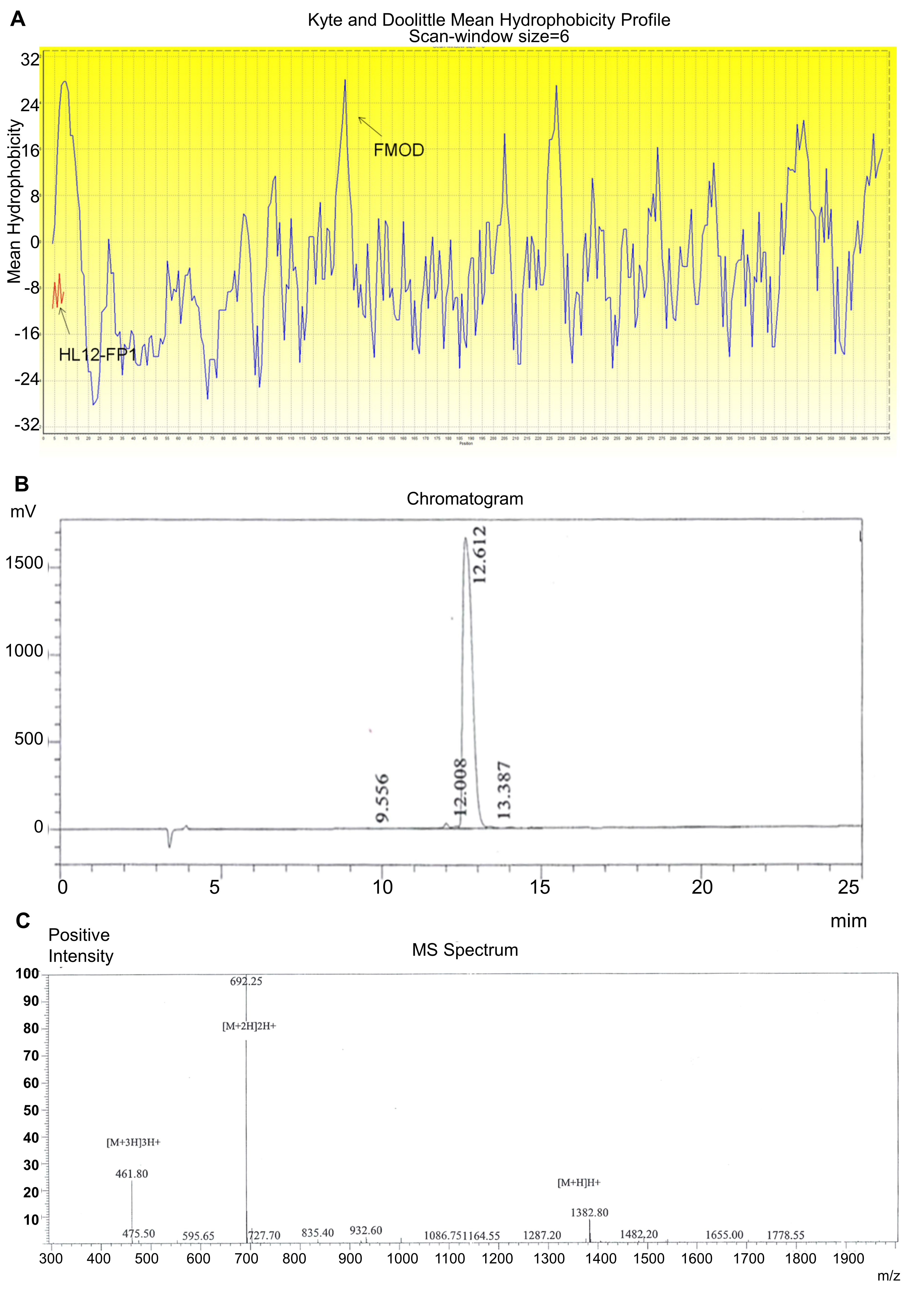

Supplement: Supplementary file 1 [file pharmaceutics-15-00944-s001.zip › SI-Figure S1 Physical property of RP4.tif]

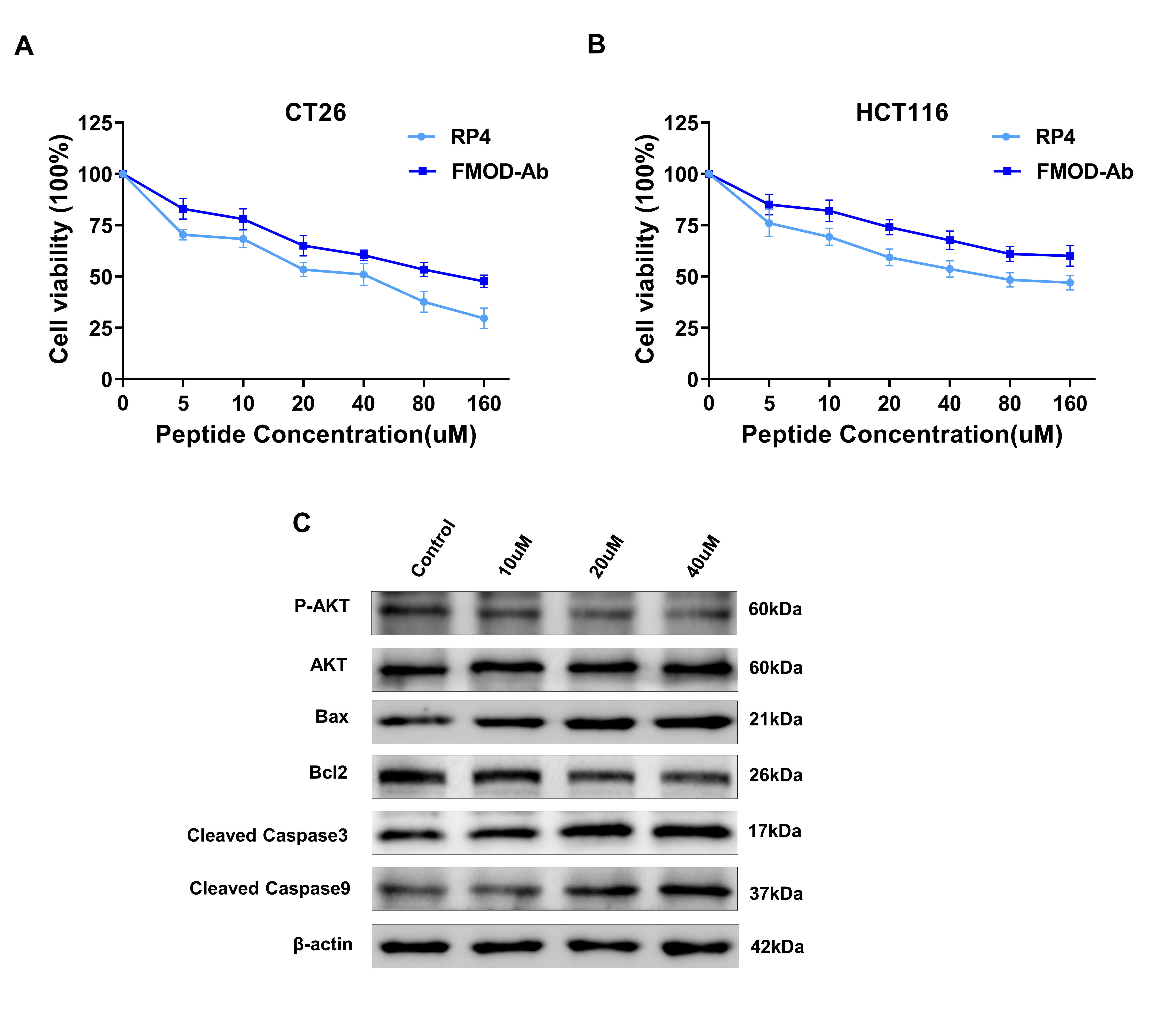

Supplement: Supplementary file 1 [file pharmaceutics-15-00944-s001.zip › SI-Figure S2 Comparison between RP4 and FMOD antibody.tif]

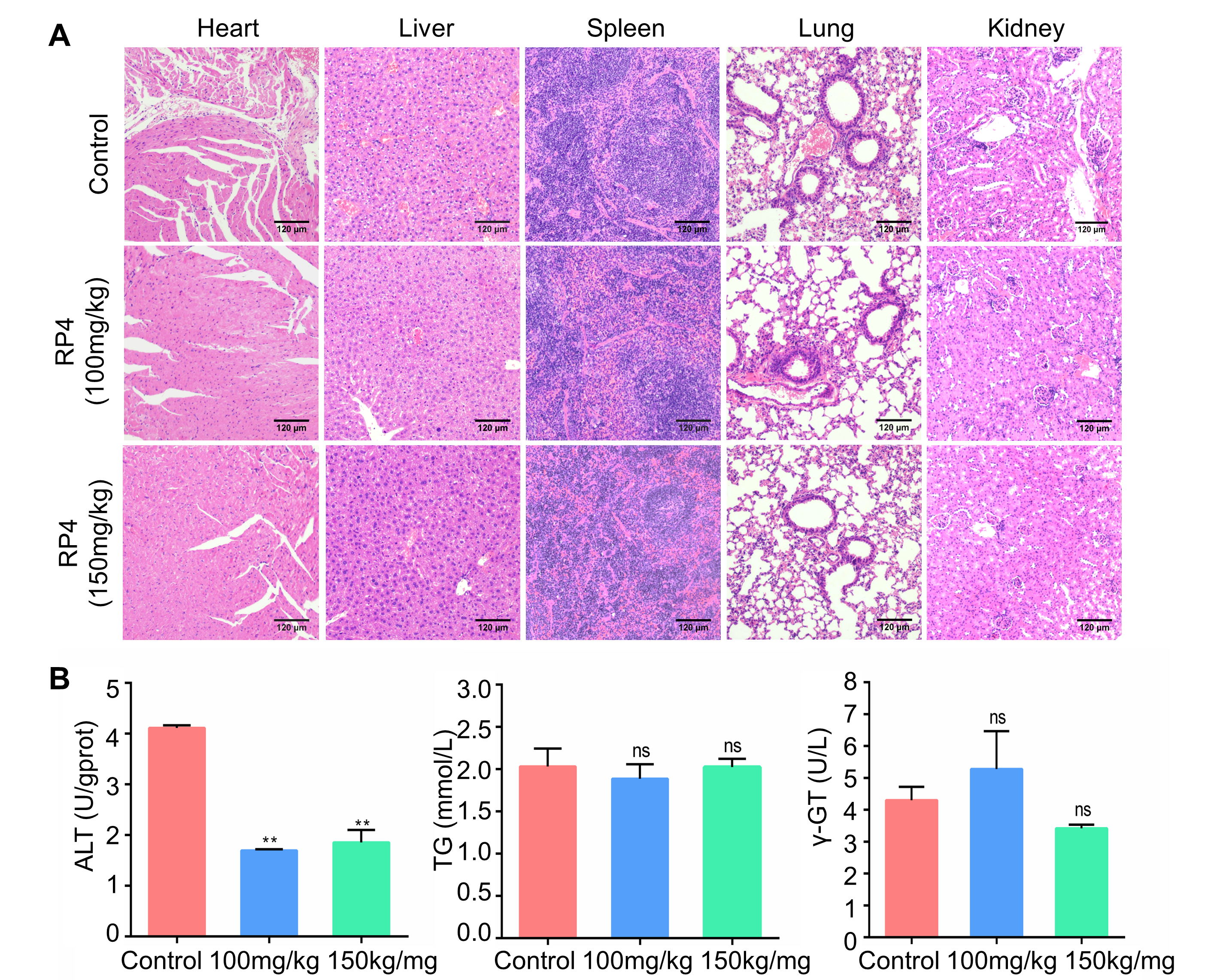

Supplement: Supplementary file 1 [file pharmaceutics-15-00944-s001.zip › SI-Figure S3 The toxicity of RP4 in vivo.tif]
